# Cardiac macrophages regulate isoproterenol-induced Takotsubo-like cardiomyopathy

**DOI:** 10.1172/jci.insight.156236

**Published:** 2022-02-08

**Authors:** Xudong Liao, Eugene Chang, Xinmiao Tang, Ippei Watanabe, Rongli Zhang, Hyun-Woo Jeong, Ralf H. Adams, Mukesh K. Jain

**Affiliations:** 1 Case Cardiovascular Research Institute, Case Western Reserve University School of Medicine, Harrington Heart and Vascular Institute, University Hospitals Cleveland Medical Center, Cleveland, Ohio, USA.; 2 Yueyang Hospital, Shanghai University of Traditional Chinese Medicine, Shanghai, China.; 3 Max Planck Institute for Molecular Biomedicine, Department of Tissue Morphogenesis, Münster, Germany.

**Keywords:** Cardiology, Cardiovascular disease, Macrophages, Monocytes

## Abstract

Takotsubo syndrome (TTS) is an acute, stress-induced cardiomyopathy that occurs predominantly in women after extreme physical and/or emotional stress. To date, our understanding of the molecular basis for TTS remains unknown and, consequently, specific therapies are lacking. Myocardial infiltration of monocytes and macrophages in TTS has been documented in clinical studies. However, the functional importance of these findings remains poorly understood. Here, we show that a single high dose of isoproterenol (ISO) in mice induced a TTS-like cardiomyopathy phenotype characterized by female predominance, severe cardiac dysfunction, and robust myocardial infiltration of macrophages. Single-cell RNA-Seq studies of myocardial immune cells revealed that TTS-like cardiomyopathy is associated with complex activation of innate and adaptive immune cells in the heart, and macrophages were identified as the dominant immune cells. Global macrophage depletion (via clodronate liposome administration) or blockade of macrophage infiltration (via a CCR2 antagonist or in CCR2-KO mice) resulted in recovery of cardiac dysfunction in ISO-challenged mice. In addition, damping myeloid cell activation by HIF1α deficiency or exposure to the immunomodulatory agent bortezomib ameliorated ISO-induced cardiac dysfunction. Collectively, our findings identify macrophages as a critical regulator of TTS pathogenesis that can be targeted for therapeutic gain.

## Graphical abstract

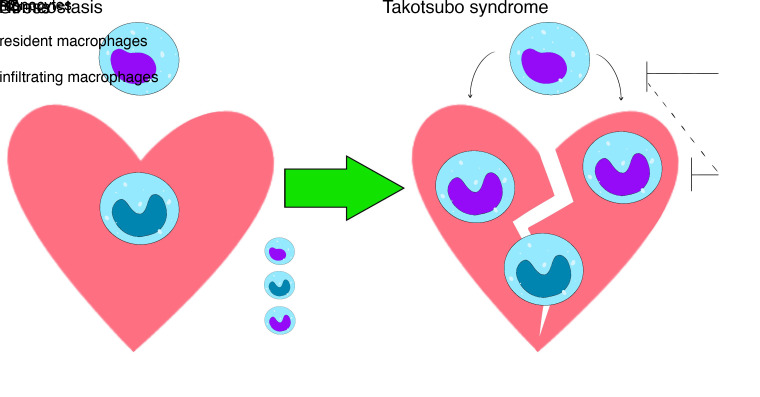

## Introduction

Takotsubo syndrome (TTS), also known as broken heart syndrome, is an acute, stress-induced cardiomyopathy that occurs after extreme physical and/or emotional stress (1). The majority (~90%) of patients with TTS are older women (aged >60 years) (2). TTS confers an in-hospital mortality rate similar to that of acute coronary syndromes (3, 4). In survivors, the recurrence rate is 2% to 4% annually up to 20% at 10 years, with a 50% rate of major adverse cardiac and cerebrovascular events despite cardiac recovery (5). TTS symptoms can be similar to those of a myocardial infarction (MI) (5), including chest pain, dyspnea, abnormal ECG (ST elevation), elevated plasma cardiac troponin level, and left ventricular wall motion abnormalities (LVWMAs). However, distinct from acute coronary syndrome, patients with TTS do not demonstrate coronary blockade and the LVWMAs do not follow a regional pattern. Therapy for TTS is largely supportive and agents such as beta blockers, which confer survival benefit in the setting of MI, are largely ineffective in patients with TTS (6, 7). To date, our understanding of the molecular basis for TTS remains unknown and, consequently, specific therapies are lacking (8).

Intense emotional and/or physical stress can trigger TTS; therefore, hyperactivation of the sympathetic nervous system (SNS) is a leading hypothesis about the mechanism of TTS (2, 8). Indeed, it has been shown that plasma levels of both epinephrine and norepinephrine were markedly increased during the acute phase of TTS to levels many times higher than that occurring with MI (5, 9), suggesting a pathogenic role of adrenergic signaling in TTS. Consistent with this idea, clinical conditions associated with high catecholamine levels, including pheochromocytoma, thyrotoxicosis, and subarachnoid hemorrhage with sympathetic storm, have been reported to cause TTS (10–12). Perhaps the most robust evidence is from cohorts of patients whose TTS was triggered by administration of β-agonists, either for medical reasons or by accident (13). Similarly, in several animal models, administration of high-dose epinephrine or isoproterenol (ISO) has been reported to induce TTS-like acute reversible left ventricular (LV) dysfunction (14).

Recent clinical studies (via biopsy specimens and MRI) have documented that TTS is characterized by a myocardial infiltration of monocytes and macrophages (15, 16). But whether these macrophages play a pathogenic role in TTS is unknown. Macrophages are found in virtually every tissue and are critical in the regulation of homeostasis and stress-induced responses. Macrophages broadly consist of 2 classes: tissue-resident macrophages and blood-borne infiltrating macrophages. At steady state, the adult heart contains 2 major subsets of Ly6C^–^/CCR2^–^ resident macrophages that originate from yolk-sac erythromyeloid progenitors (17). In addition, there are 2 minor subsets of Ly6C^+^ macrophages that are derived from hematopoietic stem cells and maintained through monocytes infiltration (18). Upon myocardial injury, cardiac macrophage numbers increase via both resident macrophage proliferation and monocyte infiltration, which is important for the progression and resolution of tissue injury (19–21). Specific markers of cardiac resident macrophages, such as Tim4 (T cell membrane protein 4, also known as TIMD4 [T cell immunoglobulin and mucin domain containing 4]) and Lyve1 (lymphatic vessel endothelial hyaluronan receptor 1), have been identified in recent studies, enabling in vivo mapping of cardiac macrophage subsets (22).

Here, we show that a single high dose of ISO induced a severe TTS-like cardiomyopathy phenotype characterized by female predominance, reversible severe cardiac dysfunction, and robust myocardial infiltration of macrophages. Using pharmacologic and genetic mouse models, we demonstrate that reducing macrophage infiltration or macrophage activation can ameliorate ISO-induced cardiac dysfunction. One of the pharmacologic agents used is an FDA-approved immunomodulatory agent; thus, these findings have immediate translational implications.

## Results

### ISO induced TTS-like cardiomyopathy in female mice.

The pathophysiology of TTS is thought to be driven by high levels of catecholamines that are released by SNS in response to extreme emotional or physical stress (5, 9). We first established a mouse model mimicking TTS by administering a single dose of ISO by i.p. injection. To assess TTS-like responses, cardiac function was assessed by determining left ventricular ejection fraction (LVEF; by transthoracic echocardiography at baseline [before injection], 1 day after injection [acute TTS], and 3, 7, and 10 days after injection [recovery]). Dose-titration studies were undertaken in female and male mice, with the primary readout being significant cardiac dysfunction. In female WT mice, a 200 mg/kg dose triggered LV dysfunction without death by day 7, whereas 400 mg/kg ISO resulted in 100% mortality after 3 days (Supplemental Figure 1A; supplemental material available online with this article; https://doi.org/10.1172/jci.insight.156236DS1). In contrast, male mice showed more variability in response to ISO (Supplemental Figure 1B). The potential reason for different TTS-like outcomes in female and male mice might be due to sex differences in sensitivity to adrenergic stimulation (23). Therefore, we used female mice administered 200 mg/kg ISO i.p. for subsequent studies. Since most patients with TTS are women (~90%), our model with female mice is consistent with the clinical setting.

**Figure 1:**
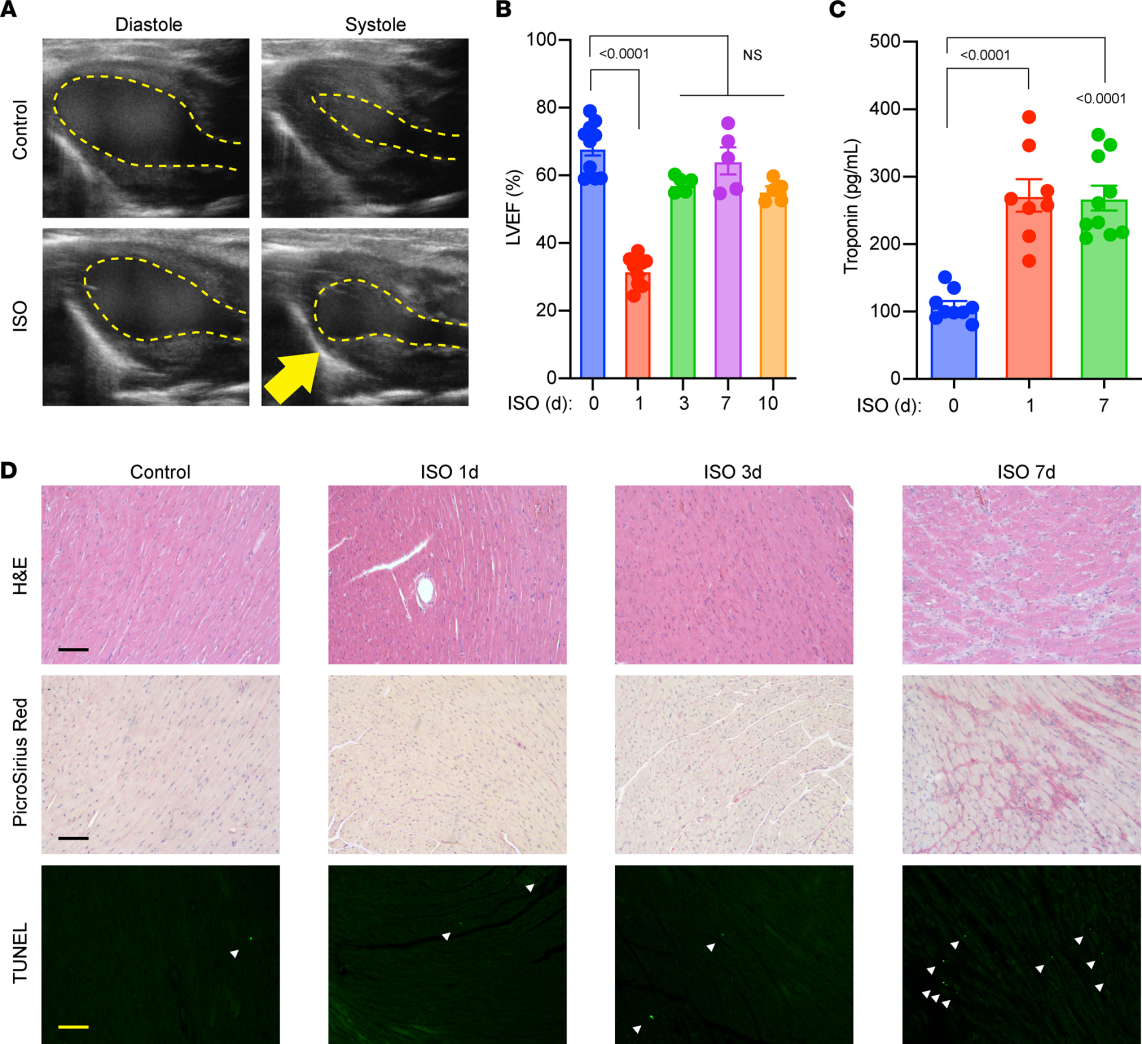
ISO-induced TTS-like cardiomyopathy in female WT mice. (**A**) Representative echocardiographic images showing apical LV ballooning. (**B**) Cardiac function in female mice (*n =* 5–10). (**C**) cTnT levels determined by ELISA. *n =* 8–10. (**D**) Histological studies with myocardial sections. Fibrosis (picrosirius red staining) and TUNEL (FITC immunofluorescence) positivity was observed from the same regions with H&E abnormality. Representative images are from 5 to 10 mice in each group. Scale bar: 50 μm. Dosing: ISO groups, 200 mg/kg ISO in PBS (i.p.); control groups: 100 μL PBS (i.p.) as vehicle. (**B** and **C**) *P* values are from 1-way ANOVA post hoc test with Tukey correction. d, days after injection.

After a single dose of ISO, female mice consistently exhibited acute LV dysfunction (LVEF <35%) at 1 day and nearly complete recovery (LVEF >60%) by 7 days (Figure 1, A and B). Of note, we confirmed ISO-induced LV apical ballooning (Figure 1A), recapitulating the classic clinical TTS phenotype. Other LV akinetic regions were also observed, with lower frequency. Consistent with clinical observations, ISO induced elevation of plasma cardiac troponin (cTnT) levels, a biomarker of myocardial injury (Figure 1C). Notably, despite LVEF recovery at 7 days after ISO administration, cTnT levels remained high, suggesting ongoing myocardial injury from a transient surge of catecholamine. Histological studies revealed sporadic regions of severe myocardial injury by day 7 after ISO injection, coupled with fibrosis and cell death in the same regions (Figure 1D). However, such injuries were not observed at earlier time points, indicating ISO-induced acute cardiac dysfunction preceded cardiac remodeling. Collectively, these findings suggest a single high dose of ISO successfully induced TTS-like cardiomyopathy in female mice.

### ISO administration induces significant myocardial infiltration of macrophages.

**Figure 2:**
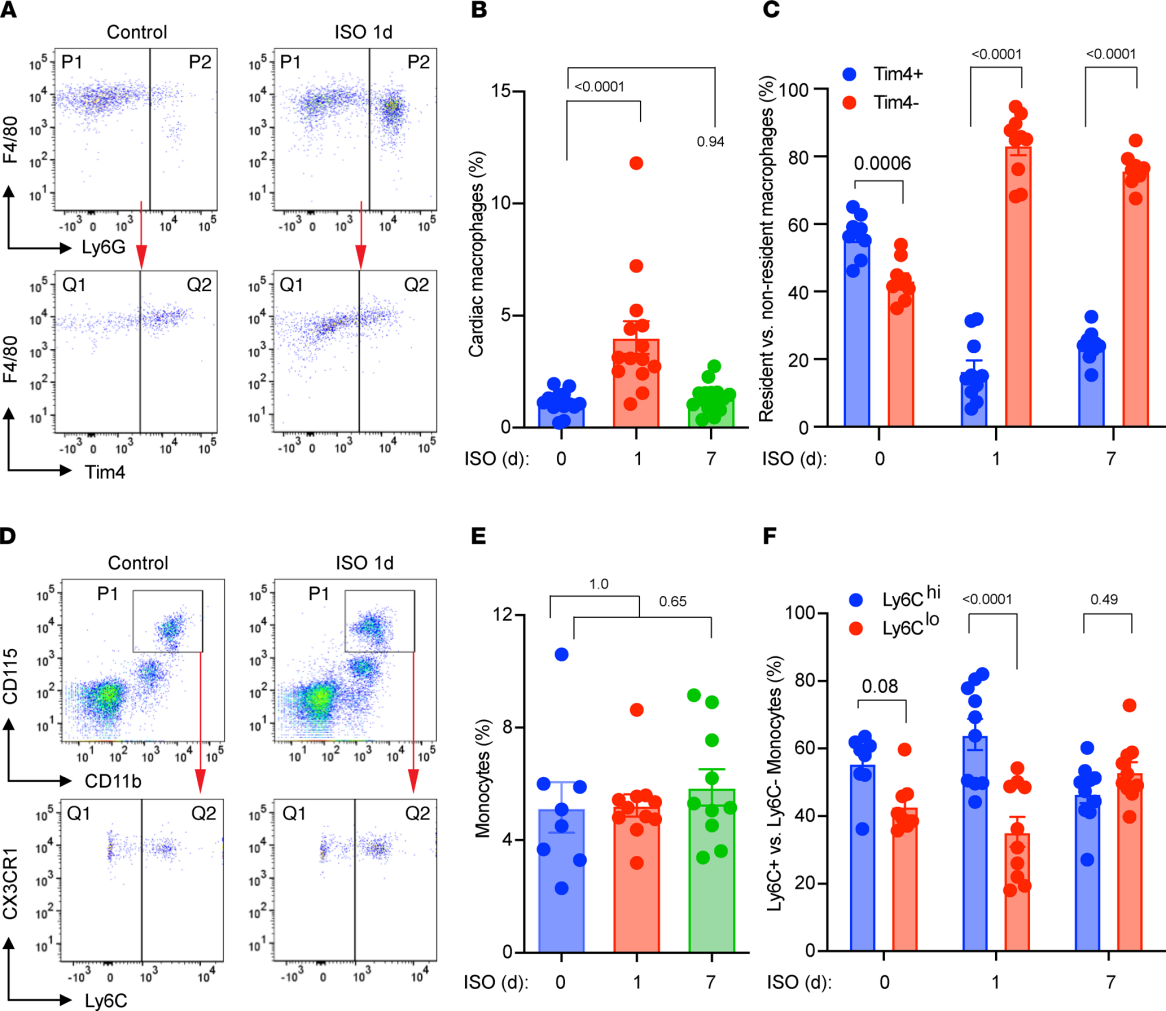
ISO-induced changes in cardiac macrophages and circulating monocytes. (**A**) Representative FACS plots of myocardial cells. P1, macrophages; P2, neutrophils; Q1, Tim4– infiltrating macrophages; Q2, Tim4+ cardiac resident macrophages. (**B**) Changes in cardiac cells after ISO treatment. The percentage of cardiac macrophages was calculated against all cells counted by flow cytometry, excluding debris. *n =* 14–16. (**C**) The percentages of Tim4^+^ and Tim4^–^ cardiac macrophage subsets (total cardiac macrophages, 100%). *n =* 9–10. (**D**) Representative FACS plots of peripheral blood monocytes. P1, monocytes (CD11b^+^CD115^hi^); Q1, Ly6C^lo^ monocytes; Q2, Ly6C^hi^ monocytes. (**E**) Total monocytes in the blood at different days after ISO treatment (all white cells, 100%). *n =* 8–11. (**F**) The percentages of Ly6C^hi^ and Ly6C^lo^ monocytes (monocytes, 100%). *n =* 8–10. Dosing: ISO: 200 mg/kg. *P* values were from 1-way ANOVA (**B** and **E**) and 2-way ANOVA (**C** and **F**) post hoc test with Tukey correction. d, days after ISO injection.

TTS myocardium is characterized with accumulation of monocytes and macrophages (15), indicating myocardial inflammation plays a role in TTS. Therefore, we subjected ISO-challenged mouse hearts to FACS to determine if ISO affected cardiac macrophage populations. Using a previously established FACS protocol (21) (Supplemental Figure 2), we found that ISO induced a significant expansion of CD45^+^CD11b^+^F4/80^+^Ly6G^–^ cardiac macrophages in the acute TTS phase, which returned to basal levels after 7 days (Figure 2, A and B).

Because cardiac macrophages can increase through self-proliferation or monocyte infiltration (21), we further investigated the source of ISO-induced macrophages in the heart. Cardiac resident macrophages express Tim4, whereas monocyte-derived infiltrating macrophages do not (22). Therefore, we analyzed surface expression of Tim4 in the macrophage population to distinguish resident versus nonresident (infiltrating) macrophage subsets. At baseline, Tim4^+^ resident macrophages were the dominant subset (21, 22). In response to ISO stimulation, the cardiac macrophages subsets underwent a shift from Tim4^+^ resident macrophages to Tim4^–^ infiltrating macrophages, suggesting that monocyte infiltration was the dominant mechanism for macrophage expansion in the ISO-stimulated heart (Figure 2, A and C). These data indicate that our model of ISO-induced mouse TTS-like cardiomyopathy recapitulated clinical TTS pathology characterized by cardiac macrophage infiltration and LV dysfunction.

Finally, we performed FACS studies with peripheral blood samples from ISO-treated mice and the control mice (Figure 2D). ISO did not trigger significant monocytosis (Figure 2E) but slightly shifted monocytes toward the proinflammatory Ly6C^(hi)^ phenotype at day 1 after injection (Figure 2F). The mild changes in circulating monocyte numbers or activation status were in sharp contrast to the significant myocardial infiltration of macrophages (Figure 2C). We speculated that ISO did not directly activate monocytes to invade and injure the myocardium. Consistent with this view, we did not observe significant proinflammatory activation of bone marrow–derived macrophages (BMDMs) treated with ISO in vitro (Supplemental Figure 3). These observations suggest that the enhanced myocardial monocyte infiltration is unlikely consequent to direct ISO-mediated monocyte activation in the periphery but rather secondary to myocardial injury. These observations may also explain the clinical observations that beta blockers did not show benefits in TTS.

### Dissecting the ISO-induced myocardial immune responses at the single-cell level.

To gain additional insights into the myocardial immune response during ISO-induced TTS-like cardiomyopathy, we sorted CD45^+^ cells from mouse hearts at 24 hours after injection (ISO vs. vehicle control), and performed single-cell RNA-Seq studies (scRNA-Seq).

**Figure 3:**
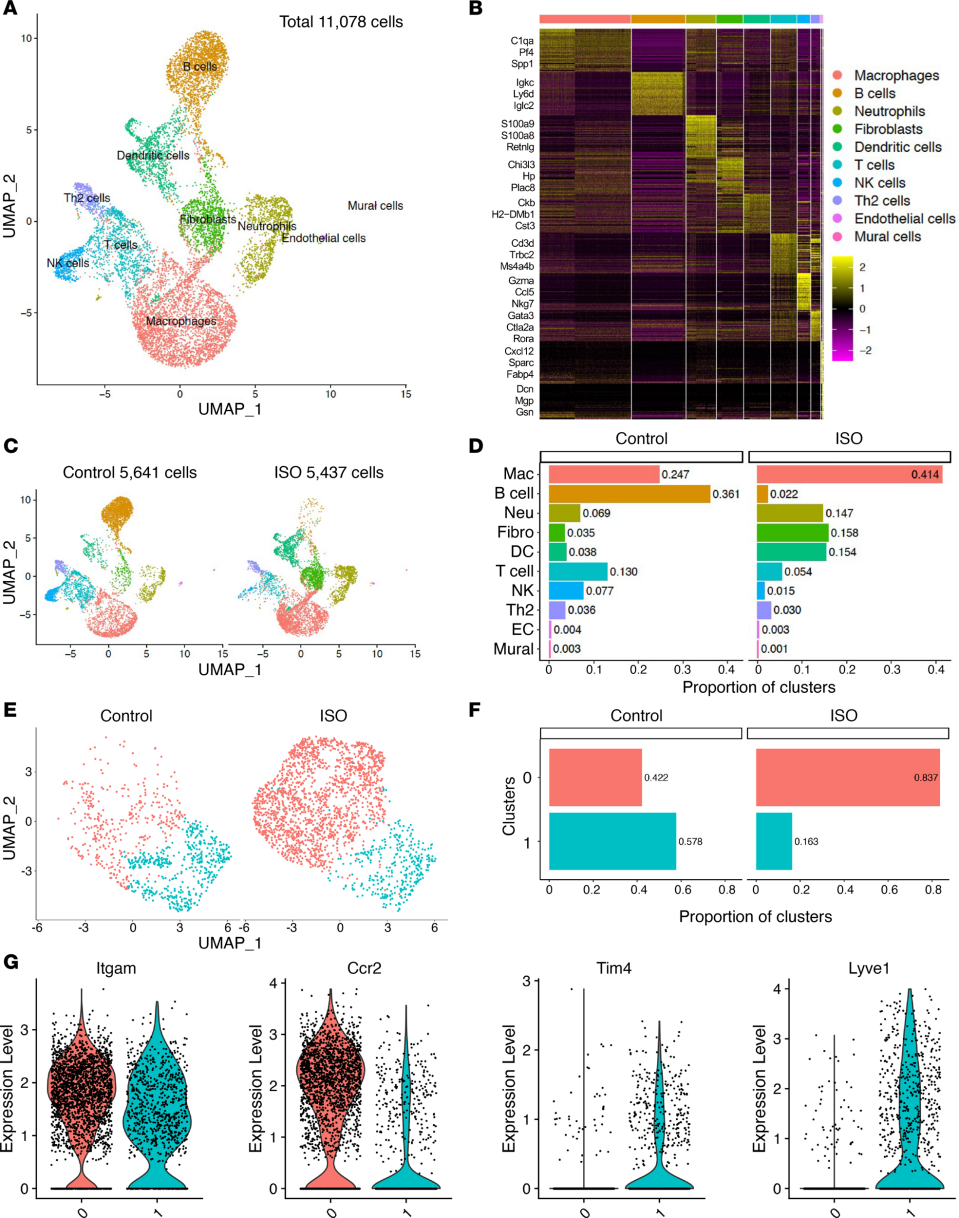
scRNA-Seq study on ISO-induced myocardial immune responses. (**A**) Ten distinct cell clusters were identified from the total of 11,078 cells. (**B**) Heatmap of the top 50 marker genes for each cluster. Selected cell type–specific markers are labeled. (**C**) UMAP of control and TTS cells. (**D**) Proportion of cell clusters before and after ISO administration. (**E**) Two subsets were identified (designated 0 and 1) from cardiac macrophages (Mac). (**F**) Proportion of Mac subclusters before and after ISO. (**G**) Violin plots showing expression of Mac marker genes in 2 subclusters. Three mouse hearts were pooled in each group (*n =* 3). Ccr2, a marker of cardiac infiltrating Macs; EC, endothelial cell; Fibro, fibroblast; Itgam, also known as Mac-1, a common marker of macrophages; Lyve1, marker of cardiac resident macrophages; Neu, neutrophil; Tim4, marker of cardiac resident macrophages.

After raw data processing and quality control, 5641 cells from the control group and 5437 cells from the ISO group were subjected to downstream bioinformatics analysis. Using Uniform Manifold Approximation and Projection (UMAP) for dimension reduction and unsupervised clustering analysis using Seurat pipeline, we identified 10 distinct cell populations from the total 11,078 cells (Figure 3A). Gene expression patterns of established canonical markers enabled us to assign putative biological identities to each cluster, namely macrophages, B cells, neutrophils, fibroblasts, DCs, T cells, NK cells, Th2 cells, endothelial cells, and mural cells (Figure 3B and Supplemental Figure 4).

Comparing the 2 groups (Figure 3, C and D), the macrophage cluster was the most abundant in the ISO group and increased approximately 2-fold compared with the control group (41.4% and 24.7%, respectively), faithfully recapitulating the monocyte–macrophage infiltration phenotype (Figure 2C). Other myeloid cells, such as neutrophils and DCs, were also increased in ISO group compared with the control group. In contrast, the lymphocyte clusters, such as B, T, Th2, and NK cells, were all reduced in the ISO group. The endothelial and mural clusters comprised a negligible percentage of cells (<0.5%) and were excluded from downstream analyses.

Because the macrophage was the most abundant cluster, we further sub-clustered it by K-nearest neighbor, graph-based, unsupervised clustering (the FindNeighbors function) and identified 2 macrophage subsets (clusters 0 and 1) that showed drastic differences between the ISO and control groups (Supplemental Figure 5A and Figure 3E). Upon ISO stimulation, there was approximately a 2-fold increase in cluster 0 and a 3-fold reduction in cluster 1 (Figure 3F). Gene expression patterns of known macrophage markers identified cluster 0 as the CCR2^+^ infiltrating macrophages and cluster 1 as the Tim4^+^Lyve1^+^ resident macrophages (Figure 3G and Supplemental Figure 5, B and C). In addition, the infiltrating macrophages (cluster 0) expressed high levels of classic inflammatory (M1) genes such as CD14 and IL1β (Supplemental Figure 5D), whereas the resident macrophages (cluster 1) expressed M2 markers such as CD163 and FOLR2 (Supplemental Figure 5E). Common macrophage genes, such as ITGAM (Mac-1), EMR1 (F4/80), and FCGR1 (CD64) were expressed in both subsets (Figure 3G and Supplemental Figure 5F). As previously reported (19), MHC-II genes (H1-Aa and H2-Ab1) were also similarly expressed in both macrophage subsets (Supplemental Figure 5G). Collectively, these clustering results are consistent with the FACS data that show ISO-induced myocardial infiltration of monocytes and monocytes further differentiated into CCR2^+^Tim4^–^ inflammatory macrophages in the myocardium.

**Figure 4:**
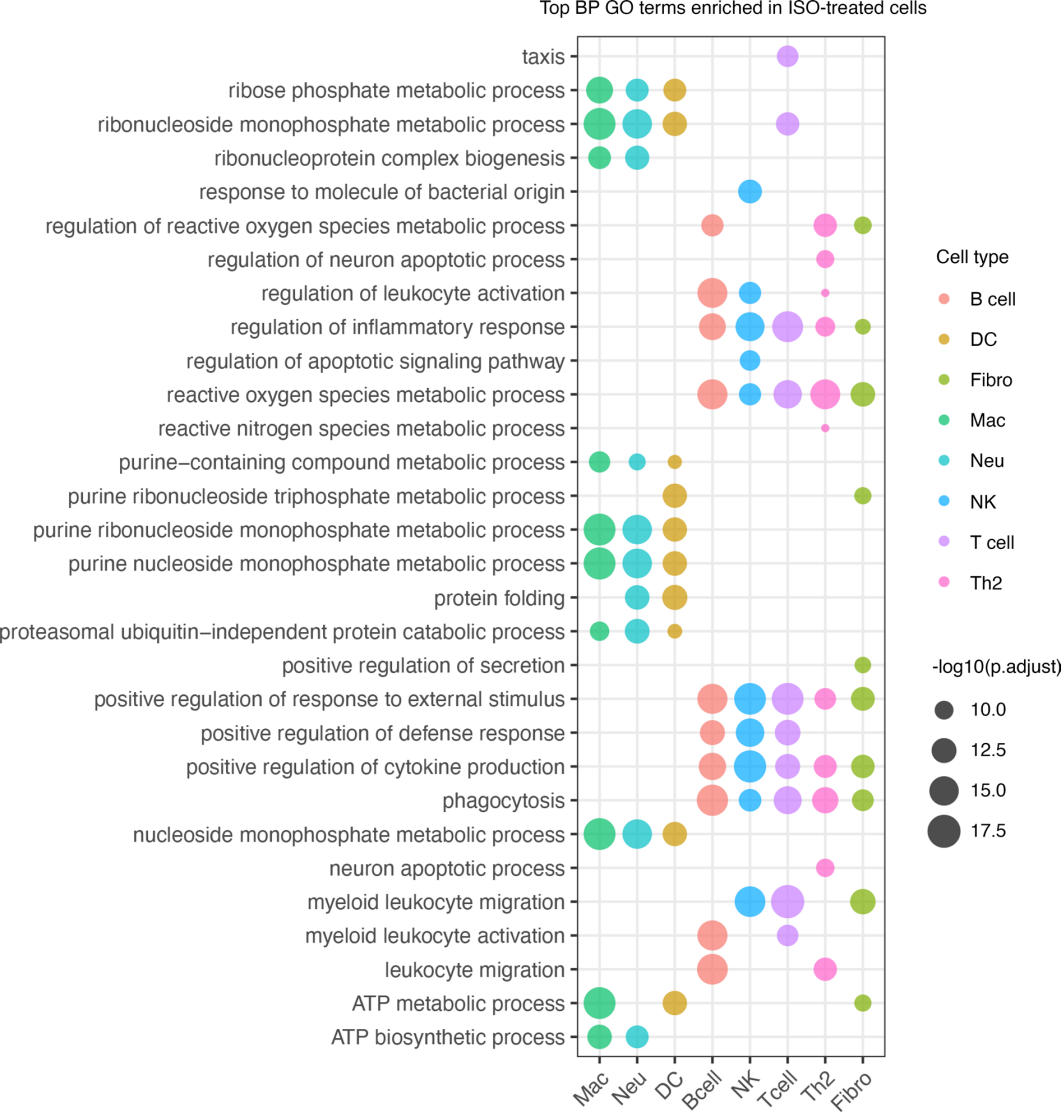
ISO-induced GO pathway enrichment in myocardial immune cells. GO analyses with DEGs that were upregulated in the ISO-treated group (TTS). Top 10 biological process (BP) GO terms according to adjusted *P* values (p.adjust) are shown. Fibro, fibroblast; Mac, macrophage; Neu, neutrophil.

Next, we performed gene ontology (GO) analysis with differentially expressed genes (DEGs; ISO vs. control, adjusted *P* < 0.05; Supplemental Figure 6). DEGS were analyzed in 2 groups: (a) genes upregulated in ISO group, and (b) genes upregulated in control group (downregulated in the TTS group). As shown in Figure 4, DEGs upregulated in the myeloid cells (i.e., macrophages, neutrophils, and DCs) from ISO group showed similar enrichment in GO terms related to energy metabolism (adenosine triphosphate biosynthesis and metabolic processes), metabolism of purine ribonucleotides and purine-containing compounds, ribose phosphate and nucleoside monophosphate metabolic processes, and ribonucleoprotein complex–related processes, indicating a robust activation status of the myeloid cells in TTS myocardium. Furthermore, purine ribonucleotides and purine-containing compounds are classic regulators of macrophage inflammation (24, 25). In contrast, DEGs upregulated in control myeloid cells (downregulated in the ISO group) were enriched in GO terms related to classic innate immune functions and were more diverse among cell types (Supplemental Figure 7). Similar GO patterns were observed in lymphocyte clusters (Figure 4 and Supplemental Figure 7). The overlapping GO terms in ISO lymphocytes were closely associated with inflammation, indicating activation of lymphocytes. The GO terms enriched in control lymphocytes were different for each cell type, concordant to their distinct functions at steady state. However, the significant reduction of lymphocyte numbers in ISO-treated mouse hearts indicates they might not be the primary regulator of TTS-like pathogenesis (Figure 3D).

### Depletion of macrophages and monocytes ameliorates ISO-induced TTS-like cardiomyopathy.

**Figure 5:**
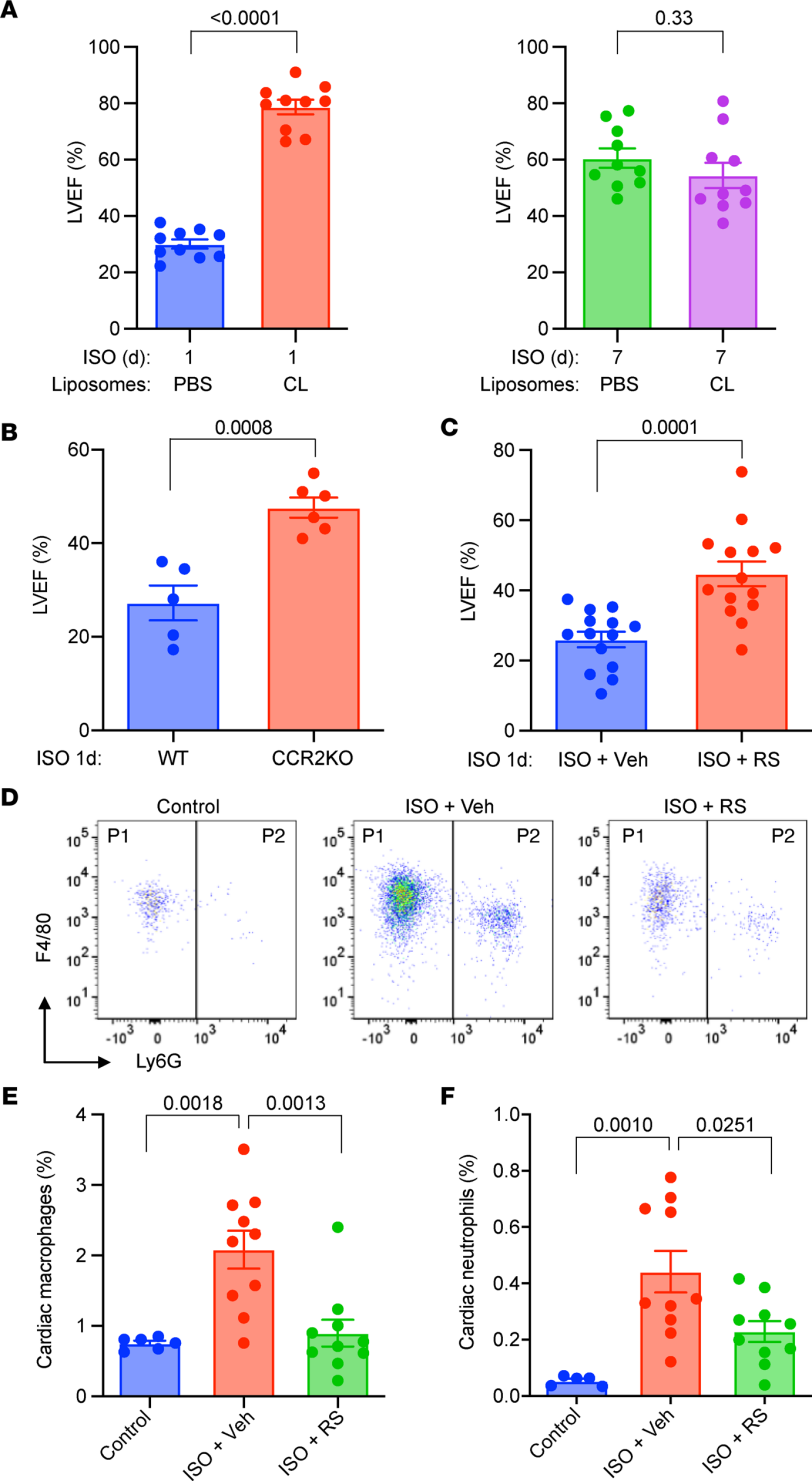
Depletion of macrophages and monocytes ameliorates TTS-like cardiomyopathy in mice. (**A**) Cardiac function in mice pretreated with clodronate-liposomes (CL) or PBS-liposome (PBS). *n =* 10. (**B**) Cardiac function in CCR2-KO and WT mice. *n =* 5. (**C**) Cardiac function in WT mice pretreated with RS-504393 (RS; 2 mg/kg twice daily by oral gavage) or PBS (Veh). *n =* 14. (**D**) Representative FACS plots of myocardial cells. Mice were pretreated with CL or RS for 3 days before ISO injection. P1, macrophages; P2, neutrophils. (**E** and **F**) Changes in cardiac macrophages (**E**) and neutrophils (**F**). *n =* 5–10. (**A**–**C**) *P* value from 2-tailed unpaired Student *t* test. (**E** and **F**) *P* values were from a 1-way ANOVA post hoc test with Tukey correction.

The significant abundance of cardiac macrophages and their increase after ISO treatment suggest these cells may play a pathogenic role in TTS-like cardiomyopathy. Therefore, we sought to target these cells to ameliorate TTS. We and others have shown that administration of clodronate-containing liposomes significantly depletes both circulating monocytes and tissue macrophages in vivo (21, 26). We found that mice pretreated with clodronate-containing liposomes (depleted of macrophages and monocytes) exhibited profound protective resistance to ISO-induced cardiac dysfunction (Figure 5A), suggesting a pathogenic role of macrophages in TTS-like cardiomyopathy.

To establish a role of monocyte infiltration, we studied TTS-like phenotypes in CCR2-KO mice, which lack circulating monocytes and are defective in chemotaxis (21). Compared with WT controls, CCR2-KO mice had significantly preserved LVEF after ISO injection (Figure 5B), conversely indicating a pathogenic role of monocytes in TTS-like cardiomyopathy. Next, we treated WT mice with RS-504393 (RS), a small molecular CCR2 antagonist (21), and found that RS administration significantly improved LVEF (Figure 5C). Accordingly, the cardioprotective effects of RS were likely due to the blockade of myeloid cell infiltration, as revealed by FACS studies (Figure 5D). Collectively, these data not only suggest a causative role of monocytes and macrophages in pathogenesis of TTS-like cardiomyopathy but suggest that targeting these cells maybe therapeutically efficacious.

### Genetic damping of myeloid activation ameliorates ISO-induced TTS-like cardiomyopathy.

**Figure 6:**
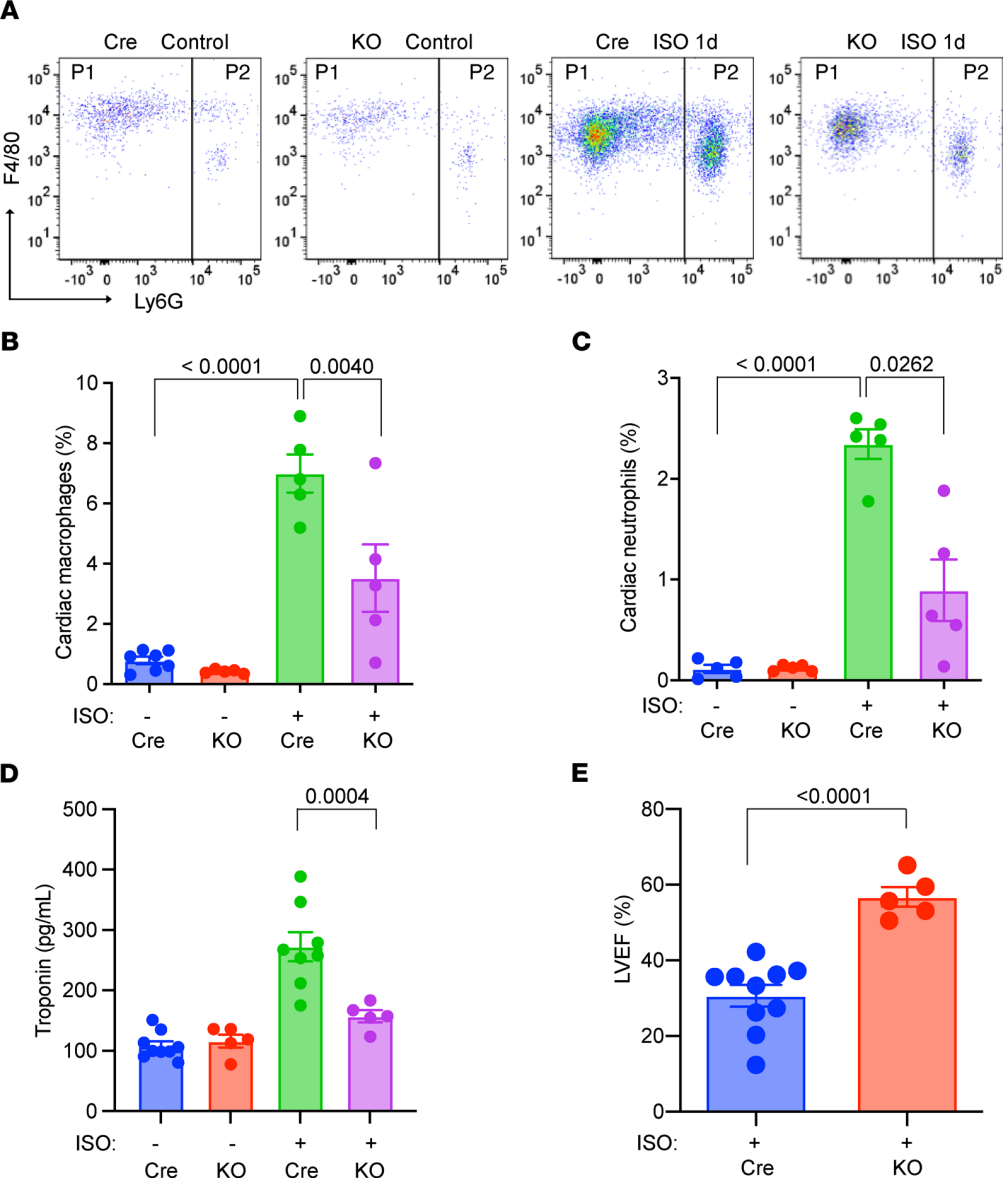
Genetic model of myeloid inhibition ameliorates ISO-induced TTS-like cardiomyopathy. (**A**) Representative FACS plots of myocardial cells from Lyz2-Cre (Cre) and Lyz2-Hif1α-KO (KO) mice. P1, macrophages; P2, neutrophils. (**B** and **C**) Changes in cardiac macrophages (**B**) and neutrophils (**C**). *n =* 5–7. (**D**) Plasma cTnT levels. *n =* 5–9. (**E**) Cardiac function in ISO-treated Cre and KO mice. *n =* 5–10. All studies were performed at day 1 after ISO. (**B**–**D**) *P* values are from a 2-way ANOVA post hoc test with Tukey correction. (**E**) *P* value is from 2-tailed unpaired Student *t* test.

It is well known that Hif1α is a critical transcription factor in myeloid cell activation (27). As such, we used Lyz2-Cre–driven myeloid Hif1α–deficient mice (Hif1α-KO) as a myeloid loss-of-function model and studied ISO-induced TTS-like cardiomyopathy. In response to ISO surge, the Hif1α-KO hearts accumulated significantly fewer macrophages and neutrophils (Figure 6, A–C), suggesting a crucial role of Hif1 signaling in TTS-associated myeloid activation. Consistently, Hif1α-KO mice also exhibited significant resistance to ISO-induced TTS-like cardiomyopathy, showing reduced plasma cTnT levels (Figure 6D) and preserved LVEF (Figure 6E). Collectively, these data demonstrate that Hif1 signaling is requisite to support myeloid activation in ISO-induced TTS-like cardiomyopathy.

### Bortezomib inhibits myeloid activation in ISO-induced TTS-like cardiomyopathy: an immunomodulatory therapy.

Given the critical role of monocytes and macrophages in ISO-induced TTS-like cardiomyopathy, we hypothesized that suppressing myeloid activation could favorably affect heart function. We tested this hypothesis with bortezomib (BZ), an FDA-approved drug. BZ was originally designed as a proteasome inhibitor that suppresses cancer cell proliferation via inhibiting proteasome-mediated degradation of tumor-suppressor proteins. We and others have shown that BZ, at very low nontumor-suppressive doses, can modulate immune cell activation (28, 29). Recent clinical studies demonstrated that BZ is an effective immunomodulatory therapy for arthritis, transplant rejection, graft-versus-host disease, and autoimmune diseases (30–34). BZ appears to be a safe immunomodulatory drug with a very favorable side effect profile (35). Here, we assessed if immunomodulatory therapy using BZ could ameliorate TTS in mice.

**Figure 7:**
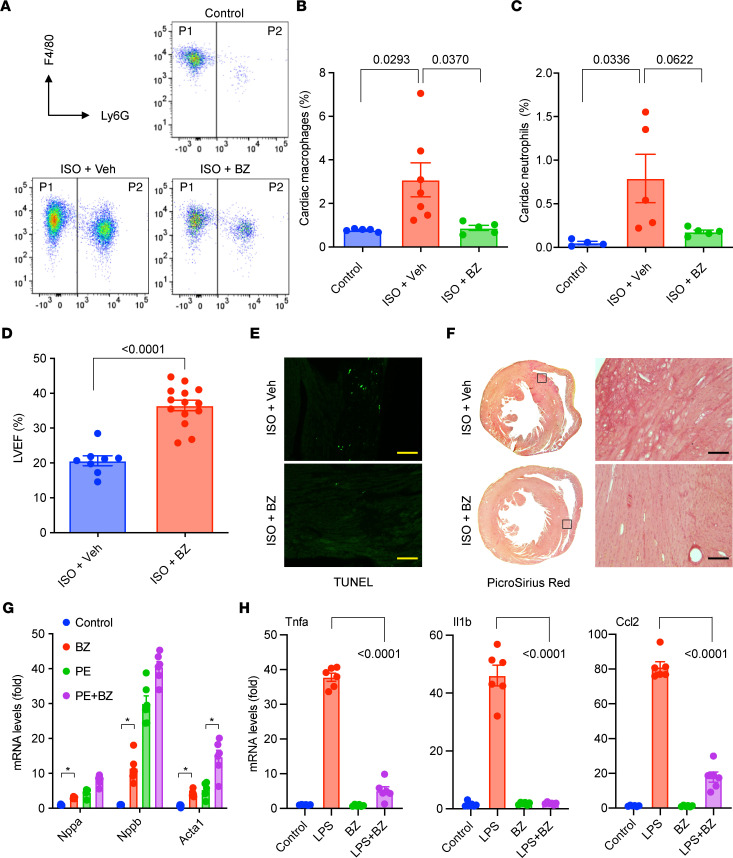
Pharmacological inhibition of myeloid activation ameliorates ISO-induced TTS-like cardiomyopathy. (**A**) Representative FACS plots of myocardial cells. P1, macrophages; P2, neutrophils. (**B** and **C**) Changes in cardiac macrophages (**B**) and neutrophils (**C**). *n =* 4–7. (**D**) Cardiac function in ISO-treated mice with or without BZ therapy. *n =* 8–14. (**E**) Cell death in the myocardium detected by TUNEL staining. (**F**) Myocardial fibrosis detected by picrosirius red staining. Boxed areas shown in high magnification on the right. Representative images are from 5 to 10 mice in each group. Scale bar: 50 μm. (**G** and **H**) Effects of BZ on cardiomyocytes (**G**) and macrophages (**H**) in vitro. *n =* 6 in each group. BZ dose, 5 μM, added 6 hours before phenylephrine (PE; 50 μM, treated for 24 hours) or LPS (50 ng/mL; treated for 24 hours). (**A**–**D**) One day after ISO administration. (**E** and **F**) Seven days after ISO administration. BZ treatment started 3 days before ISO administration (0.3 mg/kg i.p. every either day). (**D**) *P* values were from 2-tailed unpaired Student *t* test. (**B** and **C**) *P* values were from a 1-way ANOVA post hoc test with Tukey correction. (**G** and **H**) *P* values were from a 2-way ANOVA post hoc test with Tukey correction.

We first performed FACS analysis with ISO-treated and control mouse hearts and found that BZ administration significantly blocked myocardial infiltration of macrophages and neutrophils (Figure 7, A–C). Furthermore, echocardiography revealed improved LVEF in BZ-treated mice compared with the vehicle-treated (PBS) group at 1 day after ISO administration (Figure 7D). ISO-induced cardiac cell death and fibrosis by day 7 was also attenuated by BZ administration (Figure 7, E and F). Therefore, immunomodulatory therapy with BZ attenuated myeloid activation and ameliorated TTS-like pathogenesis.

Using in vitro cell culture models, we further studied the effects of BZ on cardiomyocytes and macrophages, respectively. BZ did not exhibit cardiotoxicity but had marginal hypertrophic effects on cardiomyocytes (Figure 7G). In contrast, BZ exhibited strong antiinflammatory effects in macrophages. Using LPS as a mimic of pathogen-associated molecular patterns and damage-associated molecular patterns, we treated BMDMs in vitro to trigger proinflammatory activation of macrophages. BZ significantly inhibited LPS-induced expression of proinflammatory genes, including TNF-α (*Tnfa*), IL1β (*Il1b*), CCL2 (Ccl2), Cox2 (*Ptgs2*), CD64, and Nlrp3 (Figure 7H and Supplemental Figure 8). Taken together, these data strongly support an immunomodulatory and myeloid inhibitory role of BZ in ISO-induced TTS-like cardiomyopathy.

## Discussion

TTS is a stress-induced cardiomyopathy without an effective therapy because of its unknown pathogenic mechanisms. Using a mouse model of ISO-induced TTS-like cardiomyopathy, we identified cardiac-infiltrating macrophages as critical to TTS pathogenesis. This conclusion is supported by the observation that genetic or pharmacologic approaches that prevent macrophage infiltration or activation ameliorate ISO-induced cardiac dysfunction and tissue injury. Our findings also suggest that FDA-approved immunomodulatory therapies (e.g., BZ) may be effective in reducing cardiac dysfunction and accelerating recovery.

An essential aspect of our work relates to identifying monocytes and monocyte-derived cardiac-infiltrating macrophages as critical regulators of TTS that can be therapeutically targeted. Hearts have 2 major subsets of macrophages: yolk sac–derived resident macrophages and monocyte-derived nonresident, infiltrating macrophages. Recently, studies by our group and others have revealed the distinct roles of monocytes and macrophages in the heart during acute and chronic stresses (20, 21, 36). Monocytes and monocyte-derived macrophages appear to have a proinflammatory detrimental role in the heart. In contrast, the cardiac resident macrophages appear to be antiinflammatory and cardioprotective. Clinical TTS was associated with myocardial accumulation of macrophages (15, 16), and we found the same phenotype in ISO-treated mice.

Based on the expression of multiple macrophage subset markers, such as TIM4, LYVE1, and CCR2, data from FACS and scRNA-Seq studies strongly supported ISO-induced monocyte infiltration into the myocardium, which gave rise to CCR2^+^ nonresident macrophages. These cells likely mediated myocardial proinflammatory responses, as indicated by ISO-induced enrichment in inflammation-related GO terms. Accordingly, blockade of monocyte infiltration or proinflammatory activation protected the heart from ISO-induced cardiac dysfunction. As such, these data identify monocytes and monocyte-derived infiltrating macrophages as critical regulators of TTS pathogenesis and potential therapeutic targets. However, we have not confirmed a role of cardiac resident macrophages in TTS. Given that the Tim4^+^Lyve1^+^ cardiac resident macrophages were significantly reduced after ISO challenge, it is plausible that the loss of these cells may contribute to cardiac dysfunction. Studies with inducible depletion or mutation of resident macrophages (i.e., using a Cx3cr1-based system, ref. 22) would be very interesting to explore the functions of cardiac resident macrophages in TTS.

In addition, ISO also induced expansions of neutrophils and DCs in the myocardium, despite there being fewer of these cells than macrophages. The GO terms enriched in these myeloid cells indicated they might have similar proinflammatory roles as the macrophages. More investigation is warranted of these myeloid cells. Contrary to the innate immune cells, lymphocytes were reduced in response to acute ISO challenge, indicting they likely do not drive TTS onset. Given the similar pattern in ISO-induced GO enrichment in lymphocytes, these cells appeared to be activated and might participate in cardiac remodeling after TTS. The functions of the adaptive immune system in TTS remain to be explored.

One of the limitations of this study is that we cannot precisely define a molecular regulator(s) of TTS pathogenesis. The scRNA-Seq data demonstrated complex immune responses in the ISO-treated myocardium, involving almost all immune cell types and forming a vast signaling network. The efforts to identify a specific cytokine or pathway have not been successful. However, as a complex syndrome, TTS pathogenesis may, indeed, be multifactorial. We speculate that TTS pathogenesis is an interplay between stress-injured myocardium and the immune system that responds to injury. Our data do not support ISO-induced direct activation of monocytes but suggest a response-to-injury model. The SNS signals to immune cells mainly through β2AR to mediate antiinflammatory or immunosuppressive functions (37), contradicting the proinflammatory TTS phenotype. Moreover, if myeloid cells are not activated by catecholamines, it is understandable that beta blockers cannot benefit clinical TTS because, at the point of TTS diagnosis, myocardial injury has been done and myeloid cells have been activated. The true upstream activators and downstream effectors of myeloid cells remain to be investigated and the scRNA-Seq data set would be a foundation of future studies.

Another limitation of this study is that the ISO-induced mouse cardiomyopathy model may be too simplified compared with clinical TTS, a unique and enigmatic cardiomyopathy triggered by extreme emotional or physical stress. Clearly, there is no way to model the human emotional stress in mice and the physical stress cannot be as simple as ISO surge. However, we think this mouse TTS model is practical and useful in TTS-related research. Our data are significant at 3 levels: they (1) recapitulated the transient cardiac dysfunction phenotype; (2) were associated with myocardial infiltration of macrophages; and (3) effectively worked for therapeutic models.

In a recent article, Forte et al. (38) reported that high-dose ISO induced type 2 myocardial infarction in mice, in which cardiac inflammation and fibrosis developed 2 to 8 weeks after injection. Their finding is consistent with our 1-week ISO data, likely reflecting the long-term cardiac remodeling. Instead, in the present study, we mainly focused on the acute (i.e., 1-day) TTS-like responses and showed severe cardiac dysfunction (LVEF <40%) that recovered significantly within 7 to 10 days (baseline vs. day 7 LVEF: 70% vs. 60%), similar to transient TTS. Therefore, these 2 studies share certain similarities in terms of long-term cardiac remodeling but significantly differ in the subject of investigation. Furthermore, our data demonstrated that cardiac macrophage infiltration preceded myocardial cell death and fibrosis, indicating a pathogenic role of macrophages. Finally, pharmacologically targeting monocytes and macrophages profoundly ameliorated ISO-induced TTS-like cardiomyopathy, not only confirming the causative roles of these immune cells in TTS pathogenesis but also providing targets for development of novel TTS therapies.

## Methods

Supplemental figures and tables can be found online. The scRNA-Seq data are deposited in the Gene Expression Omnibus (accession no. GSE189358).

### Animals

All mice were on a C57BL/6J background. Mice with a myeloid-specific deficiency of Hif1α (Lyz2-Cre: Hif1α^fl/fl^ [designated Hif1α-KO]) were described previously (39). The Lyz2-Cre line was used as a genetic control. WT C57BL/6J mice (WT) were purchased from The Jackson Laboratory. Mice were housed in a temperature- and humidity-controlled, specific pathogen-free facility with a 12-hour light/dark cycle and ad libitum access to water and chow.

### Cell culture

Mouse BMDMs were differentiated in DMEM containing 10% FBS and 25% L-929 conditioned medium as described previously (40). Treatment was performed in DMEM containing 10% FBS.

Neonatal rat ventricular myocytes were isolated from day-2 Sprague-Dawley rat pups and cultured as described previously (41). Treatment was performed in serum-free DMEM containing 1% insulin-transferrin-sodium selenite media supplement (Sigma-Aldrich, I1884).

### Mouse TTS model

Administration of a single high dose of ISO causes TTS-like responses in rodent models (42, 43). Female and male mice at 8 to 16 weeks of age received a single-dose i.p. injection of ISO ([−]-isoproterenol hydrochloride, Sigma-Aldrich, I6504) dissolved in 100 μL sterile Dulbecco’s PBS (Thermo Fisher Scientific, SH30028.03). The control group received an i.p. injection of 100 μL PBS as vehicle. The ISO dose was titrated between 100 and 500 mg/kg and fixed at 200 mg/kg for female mice. Cardiac function was monitored by transthoracic echocardiography before and after ISO injection for up to 10 days. For echocardiography, mice were anesthetized by inhalation of 0.1% to 0.5% isoflurane vaporized in 100% oxygen, keeping a steady heart rate above 500 bpm; LV recording was obtained in the parasternal long-axis view in B-mode on a Vevo-770 or 3100 System (Fujifilm) (41). LVEF was calculated as the percentage of LV fractional area change, using the VevoLab software. Plasma levels of cTnT were determined by a Mouse Cardiac Troponin T ELISA Kit (MyBioSource Inc., MBS726068).

### Flow cytometry

The mouse heart was perfused, excised, minced, digested with type I collagenase, mechanically disrupted, and filtered through a 70 μm cell strainer to get a single-cell suspension (21). Cells were collected by centrifugation, subjected to live-dead dye (LIVE/DEAD Fixable Yellow Dead Cell Stain Kit, Invitrogen, L34968) and surface antibody staining (fluorescent conjugated primary antibodies; BioLegend), and analyzed by flow cytometry. Cells were also sorted by flow cytometry to collect live CD45^+^ cells for transcriptomic studies. The following fluorescent antibodies were used in this study: CD45-PerCP (BD Biosciences, 561047), CD11b-AF488 (BD Biosciences, 557672), Ly6G-APC-Cy7 (BD Biosciences, 560600), F4/80-BV421 (BioLegend, 123137), and CD45-FITC (BioLegend, 103108).

### Histology

Cardiac tissue samples were fixed in 10% neutralized formalin and embedded with paraffin following standard protocols. H&E staining was perform using Mayer’s hematoxylin solution and eosin Y solution (Sigma-Aldrich, 51275, 318906). Fibrosis was stained using the Picrosirius Red Stain Kit (Polysciences, 24901) (41). TUNEL staining was performed with the ApopTag Fluorescent In Situ Apoptosis Detection Kit (Millipore, S7110) (44). Microscopic images were analyzed using NIH ImageJ software.

### RNA extraction and quantitative real-time PCR

Total RNA was purified using the Qiagen RNeasy Mini Kit, following the manufacturer’s protocol. RNA was reverse transcribed to complementary DNA using the iScript Reverse Transcription Kit (Bio-Rad, 170-8841). Quantitative real-time PCR was performed with the TaqMan method using the Roche Universal ProbeLibrary as TaqMan probes. Relative expression was calculated using the ΔΔCt method with normalization to *Gapdh*.

### Single-cell RNA-Seq studies

#### Single-cell library construction and sequencing.

CD45^+^ cells were sorted from mouse myocardium by flow cytometry. Dead cells were excluded by the live-dead dye. A total of 15,000 CD45^+^ cells were input to the 10x Genomics Chromium Controller for single-cell partitioning and Gel Bead-in-Emulsion (GEM) generation. GEMs are reaction vessels in which cDNA from each single cell is generated and uniquely barcoded. cDNA libraries were checked for quality using an Agilent 2100 Bioanalyzer. Adapters were ligated, and barcoding was accomplished for each library, followed by an additional assessment of average fragment size using the bioanalyzer. Samples were pooled and sequenced on a Novaseq 6000 S4 flow cell using the sequencing parameters outlined in the 10× Genomics Chromium Controller protocol (version CG000204).

#### Single-cell RNA-Seq analysis.

Raw sequencing data in FASTQ format were processed with UMI-tools (version 1.0.0) to generate a white list for cell barcodes and unique molecular identifiers and to extract valid sequencing reads, aligned to the mouse reference genome (mm10) with STAR (version 2.7.3a), and quantified with Subread featureCounts (version 1.6.4). Data normalization, cell clustering, and visualization were performed using Seurat (version 3.1.5). For initial quality control of the extracted gene-cell matrices, we filtered cells using the following parameters: low.threshold = 500, high.threshold = 6000 for number of genes per cell (nFeature_RNA); high.threshold = 25% for percentage of mitochondrial genes (percent.mito); and genes with parameter min.cell = 3. Filtered matrices were normalized by the LogNormalize method with a scale factor of 10,000.

Variable genes were found with parameters of selection.method = vst and nfeatures = 2000, trimmed for the genes related to cell cycle (GO:0007049), and then used for data integration (IntegrateData with FindIntegrationAnchors function), data scaling (ScaleData), and principal component analysis (RunPCA). Statistically significant principal components were determined by the JackStraw method, and the first 9 principle components were used for nonlinear dimensional reduction (UMAP) and clustering analysis (FindNeighbors) with resolution of 0.1.

#### GO terms enrichment analysis.

DEGs for each cluster between Cre and K2-KO samples were identified using the FindAllMarkers function of the Seurat package with options of only.pos = TRUE, min.pct = 0.25, and logfc.threshold = 0. Enriched GOs of molecular function, biological function, and cellular component were identified using the enrichGO function of the clusterProfiler R package with a *q* value cutoff of 0.1 and then simplified by removing redundancy of enrich terms with the simplify function with cutoff = 0.7, by = p.adjust and measure = Wang options. Top GO terms for each sample were selected by adjusted *P* values.

### Statistics

Results are presented as mean ± SEM. A 2-tailed Student *t* test was used to compare the differences between 2 groups. One-way ANOVA was used for simple multiple comparisons. Two-way ANOVA was used for studies with 2 independent variables. A post hoc test with Tukey correction was applied to multiple comparisons. Statistical significance was defined as *P* < 0.05. Statistical analyses were performed using GraphPad Prism 9 software.

### Study approval

Animal studies were approved by the Institutional Animal Care and Use Committee of Case Western Reserve University.

## Author contributions

XL, EC, XT, IW, and RZ conducted experiments and acquired and analyzed data. HWJ and RHA performed bioinformatics. XL and EC designed the research studies. MKJ supervised the project. XL and MKJ wrote the manuscript with input from all authors.

## Supplementary Material

Supplemental data

## Version 1 02/08/2022

Electronic publication
