# Supplementary material for: Cardiac macrophages regulate isoproterenol-induced Takotsubo-like cardiomyopathy
Source: JCI Insight. 2022 Feb 8;7(3):e156236. doi: 10.1172/jci.insight.156236 (PMC8855841; doi:10.1172/jci.insight.156236)
Supplement: Supplemental data [file jciinsight-7-156236-s044.pdf]

Cardiac macrophages regulate isoproterenol-induced Takotsubo-like cardiomyopathy

Xudong Liao[1, *], Eugene Chang[1, *], Xinmiao Tang[1,2], Ippei Watanabe[1], Rongli Zhang[1], Hyun Woo Jeong[3], Ralf H. Adams[3], Mukesh K. Jain[1]

1. Case Cardiovascular Research Institute, Case Western Reserve University School of Medicine, Harrington Heart and Vascular Institute, University Hospitals Cleveland Medical Center, Cleveland, Ohio 44106, USA
2. Yueyang Hospital, Shanghai University of Traditional Chinese Medicine, Shanghai 200437, China.
3. Max Planck Institute for Molecular Biomedicine, Department of Tissue Morphogenesis, 48149 Münster, Germany.

* Equal contribution

The authors have declared that no conflict of interest exists.

Correspondence:

Mukesh K. Jain, MD

2103 Cornell Rd, WRB-4522

Cleveland, OH 44106

Phone: 216.368.3607

Email: mukesh.jain2@case.edu

Supplemental figure legends

Supplemental Figure 1. ISO dose titration in female and male WT mice.
(**A**) Survival curve of female WT mice after ISO injection. ISO dose: 100, 200, 400 mg/kg. (**B**) Survival curve of male WT mice after 400 mg/kg ISO injection. Takotsubo: mice developed TTS. (**C**) LVEF in male mice after 400 mg/kg ISO injection. One way-ANOVA with Tukey correction was applied for (C) and no significance was determined. n=5-17 as indicated.

Supplemental Figure 2. FACS gating strategy.
P1: debris excluded. P1 cells were counted as total cell population. P2: dead cells excluded. P3: doublets excluded to get single cells. P4: CD45+CD11b+ population gated as myeloid cells. P5: F4/80+Ly6G- population gated as macrophages. P6: Tim4+ vs. Tim4- macrophage subsets.

Supplemental Figure 3. Effects of ISO on macrophages in vitro.
Mouse bone marrow derived macrophages were treated with ISO in vitro for indicated time. No significant induction of inflammatory genes was observed. n=3 in each group.

Supplemental Figure 4. Single-cell RNA-seq study with cardiac CD45+ cells.
(**A**) Dotplot for Top 4 marker genes for each cluster. (**B**) FeaturePlot depicting gene expression on UMAP. 10 clusters shown: Macrophages (0), B cells (1), Neutrophils (2), Fibroblasts (3), Dendritic cells (4), T cells (5), NK cells (6), Th2 cells (7), Endothelial cells (8) and Mural cells (9). In each group, 3 mouse hearts were pooled as one sample (n=3).

Supplemental Figure 5. Unsupervised clustering of macrophage subsets.
(**A**) UMAP plot showing two sub-clusters identified in cardiac macrophage population: Cluster 0 and Cluster 1. (**B-E**) Dotplot for selected macrophage marker genes in sub-clusters. (**F & G**) Violin plot for selected macrophage marker genes in sub-clusters. Expression pattern of CCR2 identified Cluster 0 as infiltrating macrophages. Expression patterns of Tim4 and Lyve1 identified Cluster 1 as resident macrophages.

Supplemental Figure 6. Single-cell RNA-seq DEGs.
Volcano plots showed DEGs in 10 major cell types identified from scRNA-seq. GO analysis was performed using these DEGs in Figure 4.

Supplemental Figure 7. GO enrichment analysis of the Control group.

Gene ontology (GO) analyses with DEGs upregulated in the vehicle-treated Control group from 8 major cell types: macrophages, neutrophils, dendritic cells, B cells, NK cells, T cells, Th2 cells, and fibroblasts. Top 10 Biological Process (BP) GO terms according to adjusted P values (p.adjust) were shown.

Supplemental Figure 8. Bortezomib (BZ) inhibited pro-inflammatory activation of macrophages in vitro.

Expression of CD64, Ptgs2 and Nlrp3 in macrophages treated with LPS, BZ, and both. Control cells were in normal medium. BZ: 5 μM, added 6h before LPS. LPS: 50 ng/ml, treated for 24h. P values were from Two-way ANOVA post-hoc test with Tukey correction. n=6 in each group.

Supplemental Table: Analyses of long-axis B-mode echocardiography

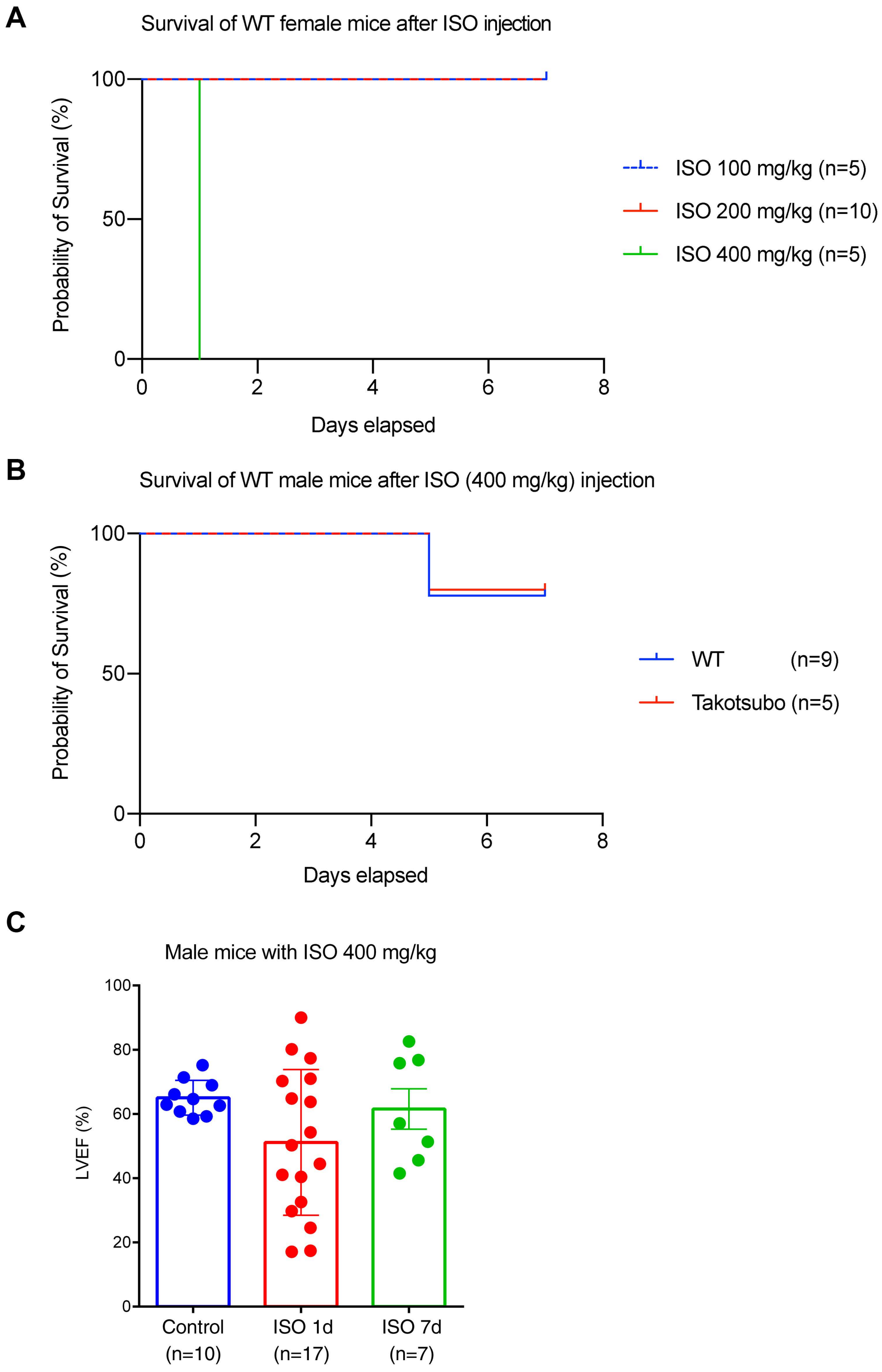

Supplemental Figure 1. ISO dose titration in female and male WT mice.
(A) Survival curve of female WT mice after ISO injection. ISO dose: 100, 200, 400 mg/kg.
(B) Survival curve of male WT mice after 400 mg/kg ISO injection. Takotsubo: mice developed TTS.
(C) LVEF in male mice after 400 mg/kg ISO injection.
One way-ANOVA with Tukey correction was applied for (C) and no significance was determined. n=5-17 as indicated.

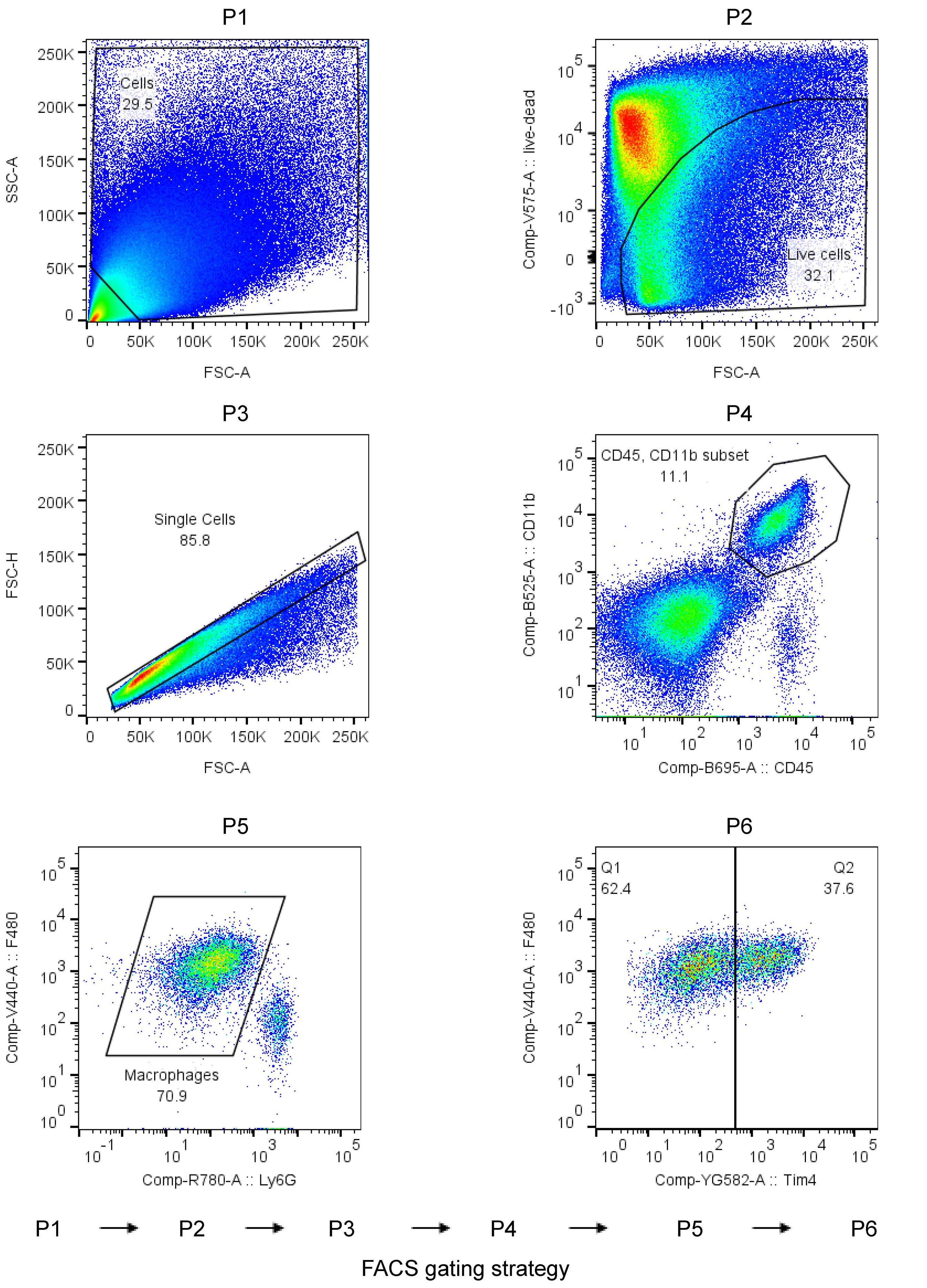

Supplemental Figure 2. FACS gating strategy.
P1: debris excluded. P1 cells were counted as total cell population. P2: dead cells excluded. P3: doublets excluded to get single cells.
P4: CD45+CD11b+ population gated as myeloid cells. P5: F4/80+Ly6G- population gated as macrophages. P6: Tim4+ vs. Tim4- macrophage subsets.

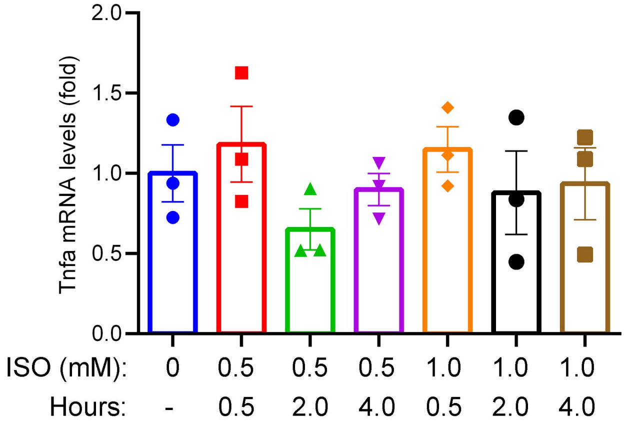

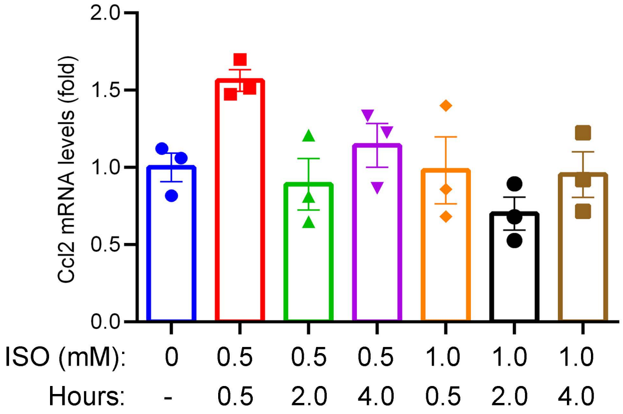

Supplemental Figure 3. Effects of ISO on macrophages in vitro.
Mouse bone marrow derived macrophages were treated with ISO in vitro for indicated time. No significant induction of inflammatory genes was observed. n=3 in each group.

A

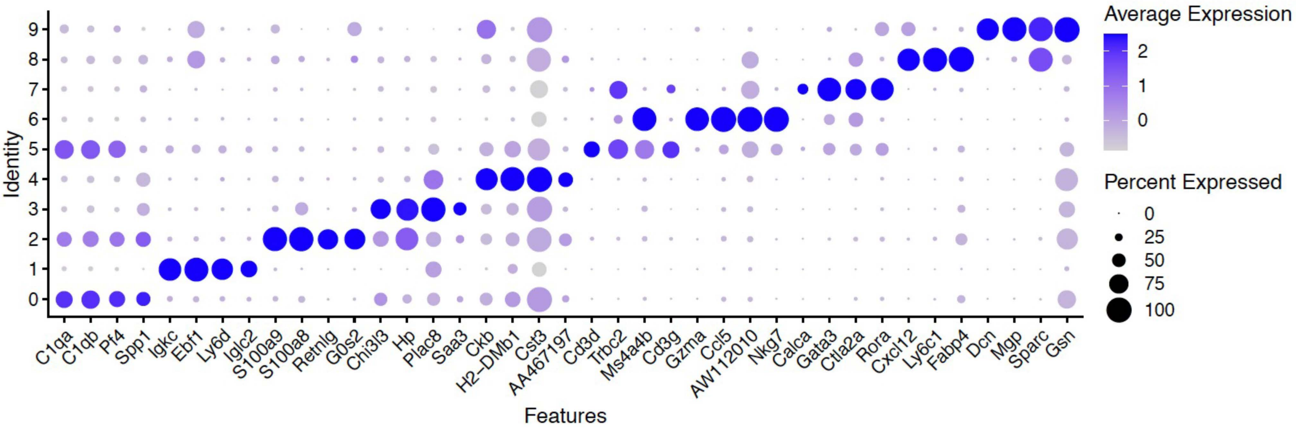

B

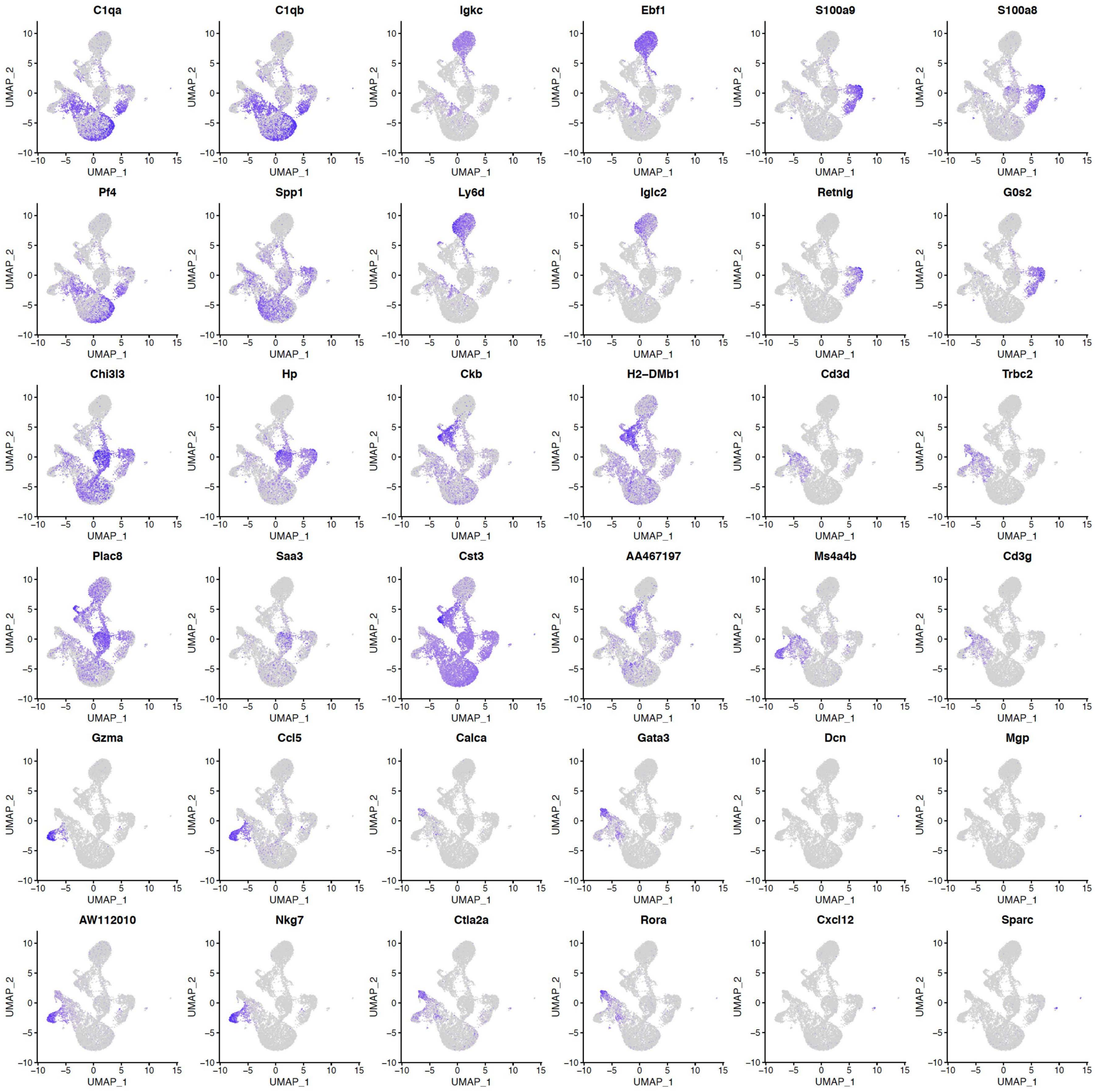

Supplemental Figure 4. Single-cell RNA-seq study with cardiac CD45+ cells.
(A) Dotplot for Top 4 marker genes for each cluster. (B) FeaturePlot depicting gene expression on UMAP.
10 clusters shown: Macrophages (0), B cells (1), Neutrophils (2), Fibroblasts (3), Dendritic cells (4), T cells (5), NK cells (6), Th2 cells (7), Endothelial cells (8) and Mural cells (9).
In each group, 3 mouse hearts were pooled as one sample (n=3).

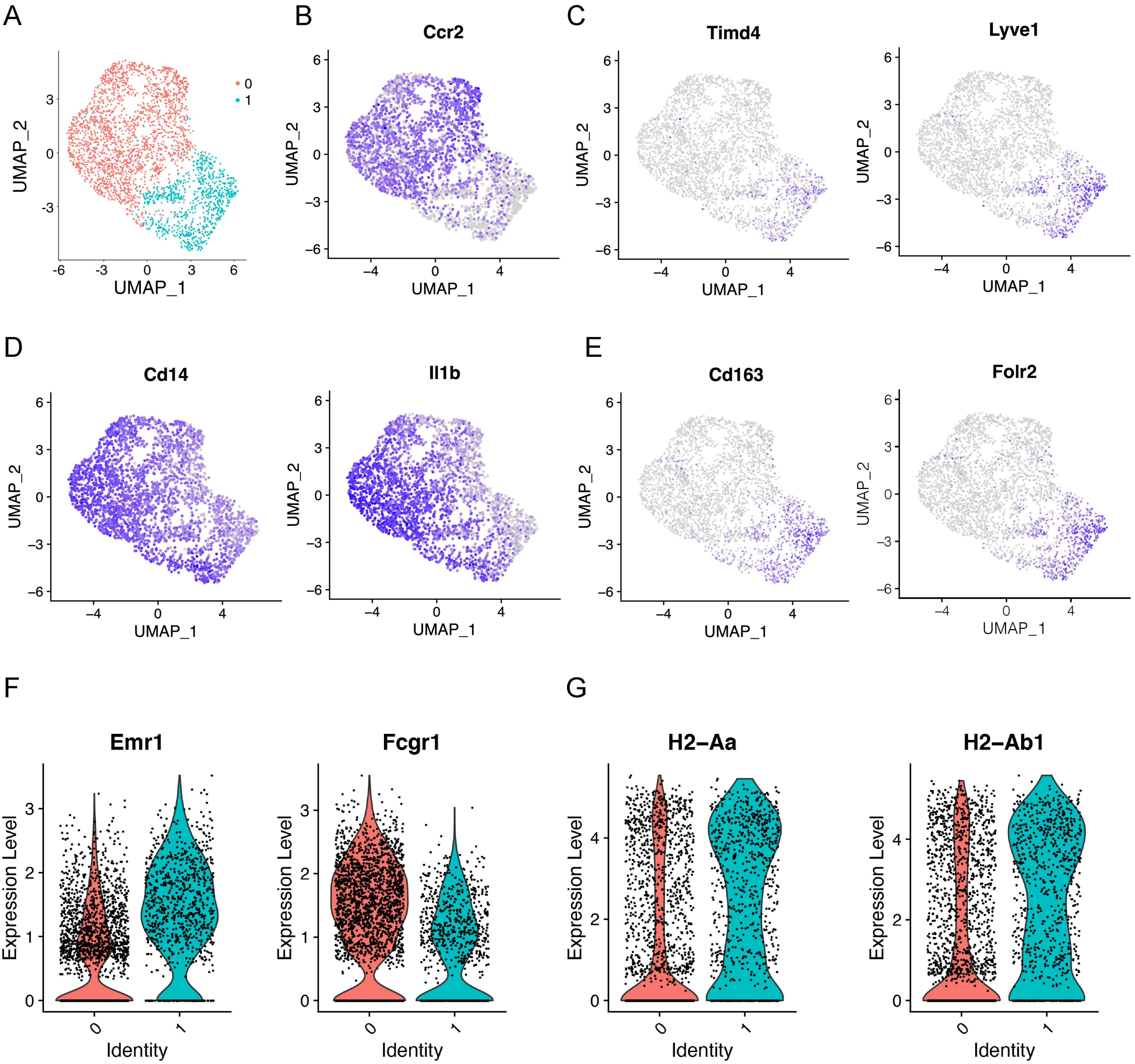

Supplemental Figure 5. Unsupervised clustering of macrophage subsets.
(A) UMAP plot showing two sub-clusters identified in cardiac macrophage population: Cluster 0 and Cluster 1.
(B-E) Dotplot for selected macrophage marker genes in sub-clusters.
(F & G) Violin plot for selected macrophage marker genes in sub-clusters.
Expression pattern of CCR2 identified Cluster 0 as infiltrating macrophages.
Expression patterns of Tim4 and Lyve1 identified Cluster 1 as resident macrophages.

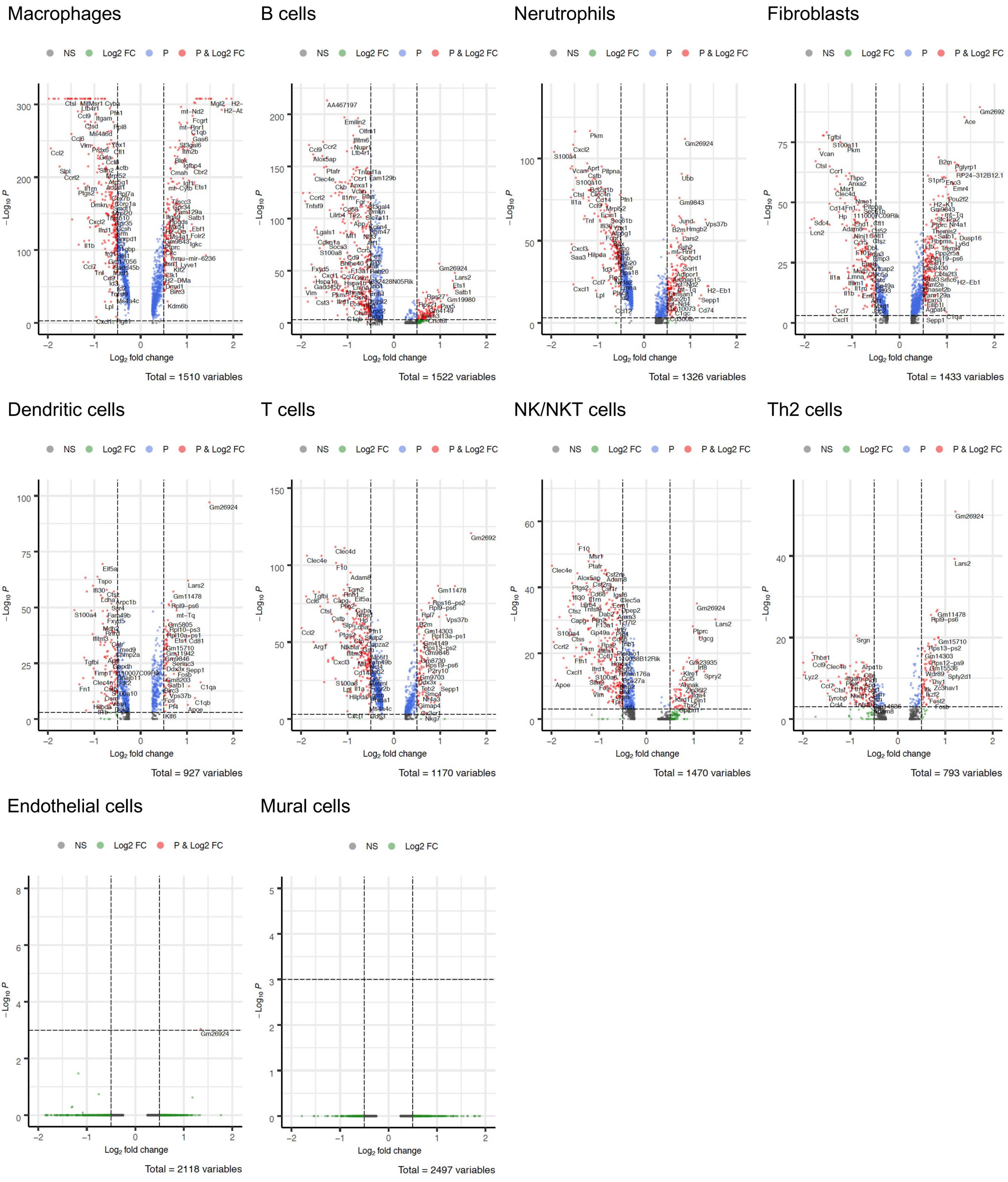

Supplemental Figure 6. Single-cell RNA-seq DEGs.
Volcano plots showed DEGs in 10 major cell types identified from scRNA-seq.
GO analysis was performed using these DEGs in Figure 4.

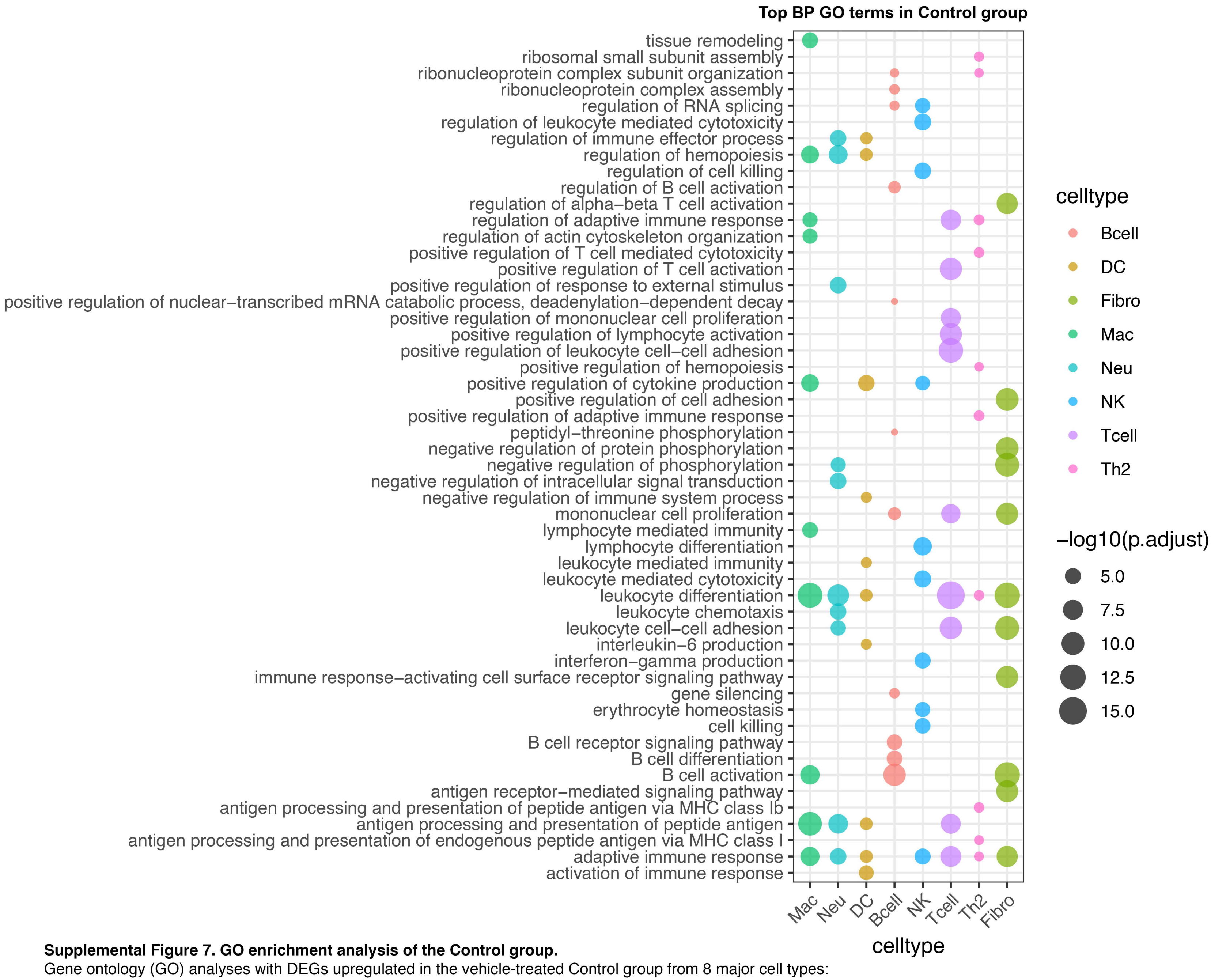

Supplemental Figure 7. GO enrichment analysis of the Control group.
Gene ontology (GO) analyses with DEGs upregulated in the vehicle-treated Control group from 8 major cell types: macrophages, neutrophils, dendritic cells, B cells, NK cells, T cells, Th2 cells, and fibroblasts.
Top 10 Biological Process (BP) GO terms according to adjusted P values (p.adjust) were shown.

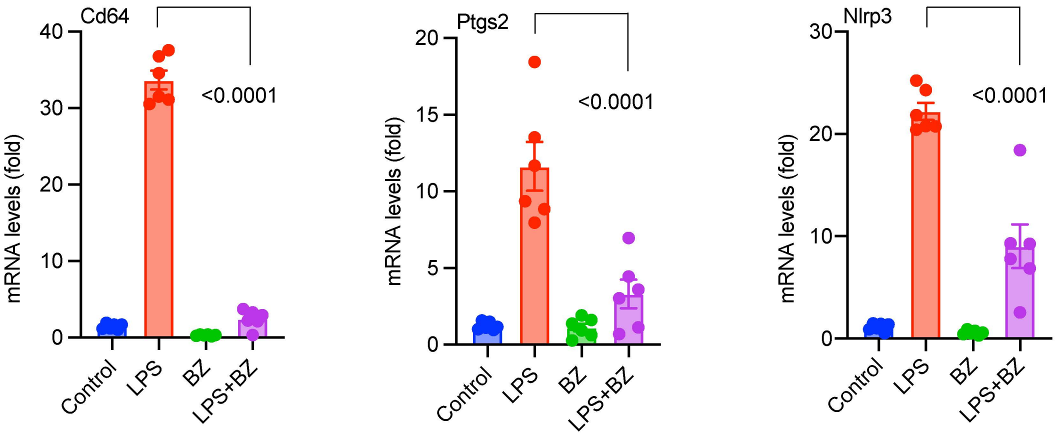

Supplemental Figure 8. Bortezomib (BZ) inhibited pro-inflammatory activation of macrophages in vitro.
Expression of CD64, Ptgs2 and Nlrp3 in macrophages treated with LPS, BZ, and both.
Control cells were in normal medium. BZ: 5 μM, added 6h before LPS. LPS: 50 ng/ml, treated for 24h.
P values were from Two-way ANOVA post-hoc test with Tukey correction. n=6 in each group.

Supplemental Table : Analyses of long-axis B-mode echocardiography

Figure 1B	ISO 0d	ISO 1d	ISO 3d	ISO 7d	ISO 10d
Endocardial Area; diastole (mm^2)	23.32 ± 3.22	25.36 ± 1.52	22.27 ± 3.66	22.59 ± 2.60	20.17 ± 3.60
Endocardial Area; systole (mm^2)	7.94 ± 1.44	16.35 ± 1.29*	9.46 ± 1.74	7.83 ± 0.72	8.91 ± 1.04
Endocardial Area Change (mm^2)	15.38 ± 1.78	9.01 ± 0.99*	12.82 ± 2.08	14.76 ± 2.24	11.26 ± 2.61
LV Fractional Area Change (%FAC)	66.11 ± 1.46	35.53 ± 3.31*	57.56 ± 2.40	64.68 ± 6.37	55.40 ± 3.10

Data shown as Mean ± SD. n=5-10, *p<0.05 vs. ISO 0d (Control), One-way ANOVA post-hoc test with Tukey correction.

Figure 5A	ISO 1d + PBS	ISO 1d + CL	ISO 7d + PBS	ISO 7d + CL
Endocardial Area; diastole (mm^2)	16.24 ± 8.10	15.50 ± 2.77	12.81 ± 6.13	15.35 ± 4.56
Endocardial Area; systole (mm^2)	11.75 ± 6.04	3.38 ± 1.65*	5.73 ± 3.30	7.46 ± 3.69
Endocardial Area Change (mm^2)	4.48 ± 2.29	12.12 ± 2.10*	7.11 ± 3.33	7.89 ± 1.59
LV Fractional Area Change (%FAC)	23.21 ± 12.19	78.70 ± 8.10*	47.51 ± 25.39	54.45 ± 14.16

Data shown as Mean ± SD. n=10, *p<0.05 vs. each PBS group, unpaired 2-tailed Student t test.

Figure 5B	WT	CCR2KO
Endocardial Area; diastole (mm^2)	22.69 ± 5.46	19.23 ± 3.02
Endocardial Area; systole (mm^2)	16.41 ± 4.24	10.14 ± 2.25*
Endocardial Area Change (mm^2)	6.28 ± 2.53	9.09 ± 1.22*
LV Fractional Area Change (%FAC)	27.27 ± 8.33	47.64 ± 5.30*

Data shown as Mean ± SD. n=5-6, *p<0.05, unpaired 2-tailed Student t test.

Figure 5C	ISO + Veh	ISO + RS
Endocardial Area; diastole (mm^2)	20.30 ± 3.22	21.38 ± 3.47
Endocardial Area; systole (mm^2)	14.09 ± 4.05	12.74 ± 2.77
Endocardial Area Change (mm^2)	6.22 ± 1.59	8.64 ± 2.22*
LV Fractional Area Change (%FAC)	26.04 ± 8.31	43.61 ± 13.43*

Data shown as Mean ± SD. n=14, *p<0.05, unpaired 2-tailed Student t test.

Figure 6E	Cre	Hif1aKO
Endocardial Area; diastole (mm^2)	17.96 ± 2.62	17.18 ± 2.69
Endocardial Area; systole (mm^2)	12.44 ± 2.27	7.54 ± 2.03*
Endocardial Area Change (mm^2)	5.52 ± 1.83	9.64 ± 0.80*
LV Fractional Area Change (%FAC)	30.67 ± 9.06	56.78 ± 5.74*

Data shown as Mean ± SD. n=5-10, *p<0.05, unpaired 2-tailed Student t test.

Figure 7D	ISO + Veh	ISO + BZ
Endocardial Area; diastole (mm^2)	21.22 ± 2.97	20.35 ± 1.68
Endocardial Area; systole (mm^2)	16.82 ± 2.39	12.91 ± 1.45*
Endocardial Area Change (mm^2)	4.40 ± 1.14	7.43 ± 1.35*
LV Fractional Area Change (%FAC)	20.65 ± 4.01	36.49 ± 5.66*

Data shown as Mean ± SD. n=8-14, *p<0.05, unpaired 2-tailed Student t test.
